# Unexplored Archaeal Diversity in the Great Ape Gut Microbiome

**DOI:** 10.1128/mSphere.00026-17

**Published:** 2017-02-22

**Authors:** Kasie Raymann, Andrew H. Moeller, Andrew L. Goodman, Howard Ochman

**Affiliations:** a Department of Integrative Biology, University of Texas at Austin, Austin, Texas, USA; b Department of Microbial Pathogenesis and Microbial Sciences Institute, Yale University School of Medicine, New Haven, Connecticut, USA; DOE Joint Genome Institute

**Keywords:** archaea, great apes, gut microbiome, microbial diversity, species interactions

## Abstract

*Archaea* are habitual residents of the human gut flora but are detected at substantially lower frequencies than bacteria. Previous studies have indicated that each human harbors very few archaeal species. However, the low diversity of human-associated archaea that has been detected could be due to the preponderance of bacteria in these communities, such that relatively few sequences are classified as *Archaea* even when microbiomes are sampled deeply. Moreover, the universal prokaryotic primer pair typically used to interrogate microbial diversity has low specificity to the archaeal domain, potentially leaving vast amounts of diversity unobserved. As a result, the prevalence, diversity, and distribution of archaea may be substantially underestimated. Here we evaluate archaeal diversity in gut microbiomes using an approach that targets virtually all known members of this domain. Comparing microbiomes across five great ape species allowed us to examine the dynamics of archaeal lineages over evolutionary time scales. These analyses revealed hundreds of gut-associated archaeal lineages, indicating that upwards of 90% of the archaeal diversity in the human and great ape gut microbiomes has been overlooked. Additionally, these results indicate a progressive reduction in archaeal diversity in the human lineage, paralleling the decline reported for bacteria.

**IMPORTANCE** Our findings show that *Archaea* are a habitual and vital component of human and great ape gut microbiomes but are largely ignored on account of the failure of previous studies to realize their full diversity. Here we report unprecedented levels of archaeal diversity in great ape gut microbiomes, exceeding that detected by conventional 16S rRNA gene surveys. Paralleling what has been reported for bacteria, there is a vast reduction of archaeal diversity in humans. Our study demonstrates that archaeal diversity in the great ape gut microbiome greatly exceeds that reported previously and provides the basis for further studies on the role of archaea in the gut microbiome.

## Abstract (precis)

Our findings show that *Archaea* are a habitual and vital component of human and great ape gut microbiomes but are largely ignored on account of the failure of previous studies to realize their full diversity. Here we report unprecedented levels of archaeal diversity in great ape gut microbiomes, exceeding that detected by conventional 16S rRNA gene surveys. Paralleling what has been reported for bacteria, there is a vast reduction of archaeal diversity in humans. Our study demonstrates that archaeal diversity in the great ape gut microbiome greatly exceeds that reported previously and provides the basis for further studies on the role of archaea in the gut microbiome.

## INTRODUCTION

*Archaea* are well-recognized residents of the human gut microbiome (1), but the prevalence, diversity, and evolution of human-associated *Archaea* are still largely unknown. Previous studies have revealed that *Methanobrevibacter* is the predominant archaeal genus in the human gut, although a few other genera, such as *Methanosphaera* and members of the order *Methanomassiliicoccales* have been recurrently detected in a small percentage of hosts (1). Most human microbiome surveys focus on the bacterial component of gut microbial communities because relatively few sequences classified as *Archaea* are recovered even when microbiomes are sampled deeply (1–6). Moreover, the universal prokaryotic primers typically used to interrogate microbial communities do not fully capture the breadth of archaeal diversity (1, 7), which has potentially left vast amounts of archaeal diversity unobserved.

Due to these limitations, we hypothesized that the archaeal diversity within the gut microbiomes of great apes is much higher than previously recognized. Using primers designed to target a broad range of archaea, we investigated the diversity of archaeal taxa within the gut microbiomes of great apes. These data revealed that upwards of 90% of the archaeal diversity in human and great ape gut microbiomes has been previously overlooked. Additionally, comparison of archaeal diversity within five great ape species revealed the dynamics of archaeal lineages across evolutionary timescales. Although several archaeal lineages have been maintained throughout hominoid evolution, paralleling what has been reported for bacteria, we observed a vast reduction of archaeal diversity in humans that has been accompanied by the loss of some associations between archaea and bacteria typical of other great apes.

## RESULTS

### Increased diversity captured with archaeon-targeted primers.

**FIG 1:**
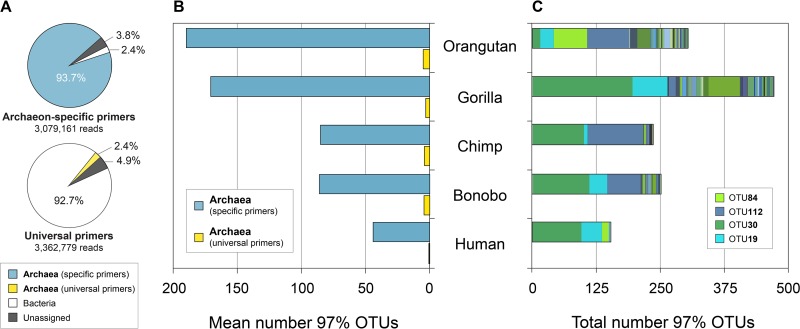
Archaeal diversity in great ape gut microbiomes. (A) Proportion of total reads assigned to archaea when gut microbiomes are surveyed with archaeon-specific primers and with the 515F/806R universal prokaryotic primers, along with percentages of bacterial and taxonomically unassigned reads. Percentages are based on the complete set of fecal samples from five great ape host species: *Homo sapiens* (*n* = 10), *Pan troglodytes* (*n* = 14), *Pan paniscus* (*n* = 18), *Gorilla gorilla* (*n* = 20), and *Pongo pygmaeus* (*n* = 8). (B) Mean numbers of archaeal OTUs when gut microbiomes are surveyed with archaeon-specific primers and with universal prokaryotic primers. Sequences were rarefied to 10,000 reads per sample, and OTUs clustered at 97% sequence identity. (C) Total numbers of archaeal OTUs when gut microbiomes are surveyed with archaeon-specific primers. Color coding of four of the most prevalent archaeal OTUs detected in great apes is indicated. Sequences were rarefied to 10,000 reads per sample, and OTUs clustered at 97% sequence identity.

We sequenced the V4-V5 region of the 16S rRNA gene using the archaeon-specific primer pair 516F/915R (see Table S1 in the supplemental material) (8, 9) to evaluate the diversity of *Archaea* in the gut microbiomes of humans (*n* = 10) and four other great ape species: *Pan troglodytes* (chimpanzee [*n* = 14]), *Pan paniscus* (bonobo [*n* = 18]), *Gorilla gorilla* (gorilla [*n* = 20]), and *Pongo pygmaeus* (orangutan [*n* = 8]). To more accurately compare diversity between species, all individuals from each species were sampled at a single geographic location: humans in the United States, chimpanzees in Tanzania, bonobos in the Democratic Republic of the Congo, gorillas in Cameroon, and orangutans in the United States. Standardization of extraction protocols enabled us to compare the diversity recovered with archaeon-specific primers to that reported for the same samples using 515F/806R primers (Table S1), which are widely used in microbiome studies (6). Of the 3,079,161 reads obtained with the archaeon-specific primers, 3.8% were bacterial in origin, which likely represents a low level of indiscriminate priming since the primers were designed to have complete specificity to *Archaea* (see Data Set S1 in the supplemental material for primer performance details). Conversely, archaeal reads accounted for only 2.4% of the 3,362,779 reads obtained with the 515F/806R universal prokaryotic primers (Fig. 1A). This low number of archaeal reads obtained with universal primers can be attributed in part to their low specificity to *Archaea* (Data Set S1). After removing unassigned reads from the archaeal and universal 16S rRNA gene data sets, the diversity within the archaeal data set was analyzed with and without bacterial reads, and all data sets were subsampled to a maximum rarefaction depth of 10,000 reads per sample. Differences in the specificity, binding preferences, and coverage spectrum of the universal prokaryotic and archaeon-specific primers prohibit direct comparisons of the relative abundance of archaea present in the two data sets. Removal of the small number of bacterial reads from the archaeal data set did not significantly change the results, so subsequent analyses are based on the data set excluding bacterial reads (see Data Set S2 for per individual read details).

10.1128/mSphere.00026-17.1 DATA SET S1  Performance of the primers used in this study. Primer performance was evaluated using TestPrime 1.0 implemented on the SILVA database (http://www.arb-silva.de/search/testprime/). For both primer pairs, the performance was calculated allowing either 0 or 1 mismatch. Coverage = (matches/eligible) × 100, specificity = 100 − (outgroup matches/outgroup matchable) × 100, Accessions = number of sequences in the taxonomic path, Eligible = number of regions with sequence data at the position of the primer pair, Mismatch = number of mismatched regions with the primer pair in the taxonomic path, and No Data = number of regions with no sequence data at the position of the primer. Download DATA SET S1, XLSX file, 0.1 MB. Copyright © 2017 Raymann et al. 2017 Raymann et al. This content is distributed under the terms of the Creative Commons Attribution 4.0 International license.

10.1128/mSphere.00026-17.2 DATA SET S2  Archaeal read counts pre- and postfiltering for each individual. Total reads for each individual using archaeon-specific primer pair Arch516F/Arch915R after quality filtering (Phred score above Q20). Shown are the percentages of chimeric sequences, singletons, unassigned, and bacterial reads detected per individual. Download DATA SET S2, XLSX file, 0.1 MB. Copyright © 2017 Raymann et al. 2017 Raymann et al. This content is distributed under the terms of the Creative Commons Attribution 4.0 International license.

10.1128/mSphere.00026-17.10 TABLE S1  Numbers of OTUs detected with (A) VSEARCH and (B) UCLUST. Download TABLE S1, PDF file, 0.1 MB. Copyright © 2017 Raymann et al. 2017 Raymann et al. This content is distributed under the terms of the Creative Commons Attribution 4.0 International license.

The mean number of archaeal operational taxonomic units (OTUs) detected in each great ape species, based on a 97% sequence identity threshold, was substantially larger when interrogating the samples with the archaeon-specific primers (orangutans, 161 OTUs for archaeon-targeted primers and 7 for universal primers; gorillas, 135 OTUs for archaeon-targeted primers and 7 for universal primers; bonobos, 71 OTUs for archaeon-targeted primers and 6 for universal primers; chimpanzees, 69 OTUs for archaeon-targeted primers and 7 for universal primers; humans, 37 OTUs for archaeon-targeted primers and 1 for universal primers) (Fig. 1B). In terms of overall counts, we identified a total of 646 archaeal 97% OTUs, with over 100 in each species (orangutans, 302; gorillas, 470; bonobos, 247; chimpanzees, 235; and humans, 120) (Fig. 1C). Even at sequencing depths of 200,000 reads, the universal primers capture less than 20% of archaeal diversity that is detected at 10,000 reads with the archaeon-specific primers (see Data Set S3 in the supplemental material). Samples were also interrogated with the universal primer pair that is commonly used to assay bacterial diversity in microbial communities (10). The mean numbers of 97% OTUs detected in each great ape species were *n* = 533 for orangutans, *n* = 330 for gorillas, *n* = 383 for bonobos, *n* = 467 for chimpanzees, and *n* = 204 for humans, consistent with bacterial diversity reported in reference 6.

10.1128/mSphere.00026-17.3 DATA SET S3  Numbers of archaeal reads and OTUs detected with the universal and archaeon-specific primer pairs. Total numbers of archaeal reads and 97% OTUs in each sample detected with universal prokaryotic primer pair 515F/806R were counted at original read depths (i.e., before rarefraction). Read depths obtained with archaeon-specific primers were rarefied to the number of archaeal reads obtained with universal prokaryotic primers (column G) prior to computing total numbers of OTUs (column J) and the percentage detected with universal versus archaeal primers (column K). Download DATA SET S3, XLSX file, 0.1 MB. Copyright © 2017 Raymann et al. 2017 Raymann et al. This content is distributed under the terms of the Creative Commons Attribution 4.0 International license.

Taxonomic assignment of 16S rRNA gene sequences are typically performed with the SILVA (11), RDP (12, 13), or Greengenes (14) databases, but these classifiers have rather limited training sets for *Archaea*. In the entire set of 646 archaeal 97% OTUs, 200 (31%) could not be classified as any genus, and 39 (6%) were classified as the incorrect phylum (e.g., assigned to phylum *Crenarchaeota* but exhibited best BLAST hits to members of the phylum *Thaumarchaeota*). Because assignment of archaeal OTUs to known taxa is problematic, we analyzed the phylotypic diversity without relying on a reference-based taxonomy and instead base our analyses on 94% OTUs and 97% OTUs, which have traditionally been considered sequence thresholds that delineate genera and species, respectively (15).

At 97% clustering, 74 OTUs were common to all five great ape species (see Fig. S1A in the supplemental material). When considering only OTUs constituting the core archaeal microbiome (defined as those OTUs present in at least 80% of the individuals in each host species), nine 97% OTUs are shared by all species. The core archaeal microbiome comprises only 9% of the total number of 97% OTUs detected in gorillas but 26% of the archaeal diversity in orangutans (Fig. S1A and B), indicating that there is no simple relationship between the total diversity harbored by a species and the scope of its core microbiome.

10.1128/mSphere.00026-17.6 FIG S1  Number of archaeal 97% OTUs shared among great ape species. (A) Venn diagram showing the number of archaeal 97% OTUs shared between species, with the full number of archaeal 97% OTUs detected in each host species shown under the species name. (B) Venn diagram showing the number of core archaeal 97% OTUs shared between species, with the full number of core OTUs detected in each host species shown under the species name. Download FIG S1, PDF file, 0.1 MB. Copyright © 2017 Raymann et al. 2017 Raymann et al. This content is distributed under the terms of the Creative Commons Attribution 4.0 International license.

Phylogenetic analysis of the core archaeal OTUs from each species (incorporating one representative sequence per OTU) revealed two lineages—OTU30 and OTU84—that could be unequivocally assigned to the known human gut associates *Methanobrevibacter smithii* and *Methanosphaera stadtmanae* (see Fig. S2 in the supplemental material). The 97% OTU corresponding to *Methanobrevibacter smithii* (OTU30) is present in all species sampled and occurs at the highest relative abundance in a majority of the humans (Fig. 1C; see Data Set S4 in the supplemental material). In addition to *Methanobrevibacter smithii*, *Methanomassiliicoccales* sp. (OTU112) and *Methanobrevibacter* sp. (OTU19) were present in all humans sampled, and *Methanosphaera stadtmanae* was present in 90% of humans sampled. Despite the extensive OTU diversity detected with archaeon-specific primers, none of the OTUs classified as the order *Methanomassiliicoccales* matched the reference species typically detected in the human microbiome (Fig. S2). Although many of the 14 core archaeal OTUs in the human gut microbiome are present in at least one other ape species (Fig. S1B), it is not possible to infer their gain or loss during great ape diversification because several are shared exclusively by paraphyletic taxa or are only sporadically present in other species.

10.1128/mSphere.00026-17.4 DATA SET S4  Prevalence of archaeal OTUs in the great ape gut microbiome species. The nine core 97% OTUs present in all great ape host species are shown in gray. The core 97% OTUs of each species (i.e., those present in >80% of sampled individuals) are in black boldface (related to Fig. S2). Download DATA SET S4, XLSX file, 0.1 MB. Copyright © 2017 Raymann et al. 2017 Raymann et al. This content is distributed under the terms of the Creative Commons Attribution 4.0 International license.

10.1128/mSphere.00026-17.7 FIG S2  Phylogenetic diversity of archaeal 97% OTUs. Unrooted maximum likelihood phylogeny of the 110 archaeal core 97% OTUs is shown. The phylogeny was inferred by PhyML (GTR+Γ4) and is based on a representative sequence for each OTU. OTUs included in the analysis were present in >80% of individuals from each species, and the nine OTUs present in all great ape host species are indicated in red. The scale bar represents average number of substitutions per site. The reliability for the internal branches of the ML tree was assessed by the bootstrapping method (100 bootstrap replicates) and the approximate likelihood ratio test (aLRT–SH-Like) (51). Dots at nodes denote aLRT values: black, >0.8; gray, >0.5; white, <0.5. Download FIG S2, PDF file, 0.1 MB. Copyright © 2017 Raymann et al. 2017 Raymann et al. This content is distributed under the terms of the Creative Commons Attribution 4.0 International license.

### Declining archaeal diversity in the gut microbiome during great ape diversification.

**FIG 2:**
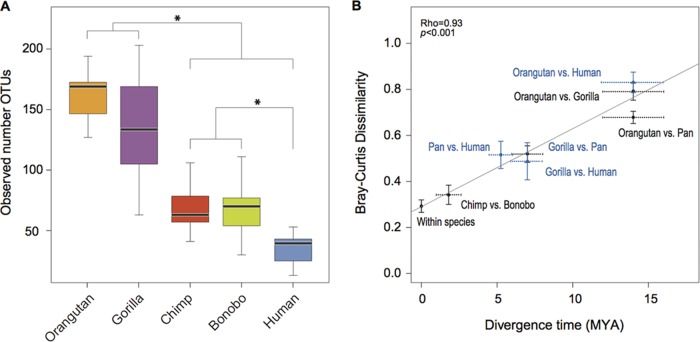
Changes in archaeal diversity during great ape diversification. (A) Alpha diversity of archaeal OTUs in great ape gut microbiomes. Box-and-whiskers plots show high, low, and median values, with the lower and upper edges of each box denoting the first and third quartiles, respectively. Species comparisons yielding statistically significant differences are indicated by asterisks (*, *P* < 0.05, Wilcoxon rank-sum test after Bonferroni correction). (B) Association between microbiome divergence, indexed by Bray-Curtis dissimilarity of 97% OTUs, and host divergence time (Spearman correlation; rho = 0.93, *P* < 0.001). Black crosses denote comparisons within all species and between species, excluding humans, and blue crosses denote comparisons between humans and other great ape species. Capped verticals of each cross denote the 99% confidence interval of pairwise Bray-Curtis dissimilarities, and the dotted horizontal lines show the range of divergence times estimated for the species pair considered. A significant correlation between Bray-Curtis dissimilarities and divergence times was also ascertained by a Mantel test (*P* < 0.01, 10,000 replicates).

We compared the numbers of 97% OTUs recovered from each of the five host species to determine if archaeal diversity within the gut microbiome has changed over the evolutionary history of great apes: phylogenetic relationship of great apes = ({[human (chimp, bonobo)], gorilla}, orangutan). Paralleling what has been reported for bacteria (6), archaeal diversity is lower in humans than in other great apes (Fig. 2A; see Fig. S3A in the supplemental material). More than 90% of the archaeal 97% OTUs detected in humans, chimpanzees, and bonobos were detected in one or more of the other great ape species, whereas gorillas and orangutans harbor many unique OTUs (Fig. S1A). Principal component analysis of both unweighted and unweighted UniFrac distances differentiated host genera, but the two species of *Pan* broadly overlapped in archaeal diversity (Fig. S3B and C). Because the two* Pan* species are from geographically separated locations—bonobos reside in the Democratic Republic of the Congo and chimpanzees in Tanzania—the similarities in their microbiome compositions are a not consequence of shared habitats.

10.1128/mSphere.00026-17.8 FIG S3  Diversity of archaeal 97% OTUs in the gut microbiomes of great apes. (A) Diversity measured by Shannon’s index, Simpson’s index, Fisher’s alpha, and Chao1. Box-and-whiskers plots show high, low, and median values, with the lower and upper edges of each box denoting the first and third quartiles, respectively. Species comparisons yielding statistically significant differences are indicated by asterisks (*, *P* < 0.05, Wilcoxon rank-sum test after Bonferroni correction). (B) Principal coordinate analysis of unweighted UniFrac distances of the archaeal component of the gut microbiomes of five great ape species. (C) Principal coordinate analysis of weighted UniFrac distances of the archaeal component of the gut microbiomes of five great ape species. Download FIG S3, PDF file, 0.1 MB. Copyright © 2017 Raymann et al. 2017 Raymann et al. This content is distributed under the terms of the Creative Commons Attribution 4.0 International license.

Despite differences among species in diet, habitat, and lifestyles, it has been shown that the bacterial composition of the gut microbiome has changed at a relatively clock-like rate during the diversification of great apes (6). The same is true for *Archaea*: for 97% OTUs, the Bray-Curtis dissimilarity between the archaeal communities of each pair of species increases linearly with the divergence times between species (*r*^2^ = 0.93, *P* = 0.0001) (Fig. 2B).

**FIG 3:**
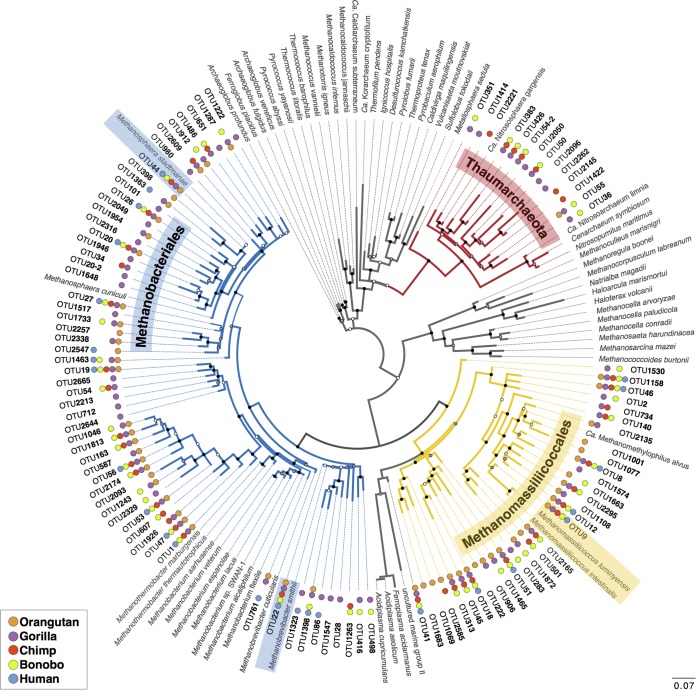
Phylogenetic diversity of archaeal lineages in the great ape gut microbiome. Shown is an unrooted maximum likelihood tree of 102 archaeal 94% OTUs detected in great apes (numbered OTUs) and 58 archaeal 16S reference sequences (Latin binomes). Phylogeny was inferred by PhyML (GTR+Γ4). Colored branches denote the major three archaeal groups to which all identified OTUs belong: *Methanobacteriales* (blue), *Methanomassiliicoccales* (yellow), and *Thaumarchaeota* (red). OTUs identical or with very high similarity to archaeal strains previously identified in the human gut microbiome are highlighted: *M. smithii* and *M. stadtmanae* in blue and *M. luminyensis* and “*Ca*. Methanomassiliicoccales alvus” in yellow. Solid circles at branch termini show the OTU distribution among host species, with OTUs considered present if found in any individual of a species. The scale bar represents the average number of substitutions per site. Reliability of internal branches was assessed by bootstrapping (100 replicates) and the approximate likelihood ratio test (aLRT–SH-Like). Dots at nodes denote aLRT values: black, >0.8; gray, >0.5; white, < 0.5.

When examined at the level of 94% OTUs, the archaeal diversity within the great ape microbiomes shows patterns consistent with those detected with 97% OTUs—most notably, there is a decrease in diversity in humans (see Fig. S4 in the supplemental material). Overall, the great apes harbored a total of 104 archaeal 94% OTUs at a rarefaction depth of 10,000 reads. Phylogenetic analysis of one representative of each of these 104 OTUs with 58 reference archaeal 16S rRNA gene sequences (i.e., well-classified representatives from the major archaeal lineages with complete genomes) placed most of the 94% OTUs in the orders *Methanobacteriales*, *Methanomassiliicoccales*, and *Nitrososphaerales* (Fig. 3). Members of these orders have previously been detected in the human gut microbiome (1), but as noted above, only *Methanobrevibacter* is reported to be a habitual resident.

10.1128/mSphere.00026-17.9 FIG S4  Diversity of archaeal 94% OTUs in the gut microbiomes of great apes. Diversity was measured by the following indices: observed OTUs, Shannon’s index, Simpson’s index, Fisher’s alpha, and Chao1. Box-and-whiskers plots show high, low, and median values, with the lower and upper edges of each box denoting the first and third quartiles, respectively. Species comparisons yielding statistically significant differences are indicated by asterisks (*, *P* < 0.05, Wilcoxon rank-sum test after Bonferroni correction). Download FIG S4, PDF file, 0.1 MB. Copyright © 2017 Raymann et al. 2017 Raymann et al. This content is distributed under the terms of the Creative Commons Attribution 4.0 International license.

Mapping the phylogenetic distribution of 94% OTUs in each host species revealed that there is much more diversity of *Methanobacteriales*- and *Methanomassiliicoccales*-related OTUs in humans than has been previously recognized. The 94% OTUs clustering within the phylum *Thaumarchaeota* (order *Nitrososphaerales*) were recovered exclusively from the nonhuman apes (Fig. 3). Moreover, not one 94% OTU is uniquely present in humans, whereas the nonhuman great ape species, particularly gorillas and orangutans, each possess many unique 94% OTUs. Most 94% OTUs could not be assigned to a prescribed archaeal genus, and the phylogenetic distance between the OTUs that we recovered and the reference genomes demonstrates that there are vast amounts of archaeal diversity in the great ape microbiome that have not previously been observed (Fig. 3).

### Microbial associations maintained throughout hominoid evolution.

**FIG 4:**
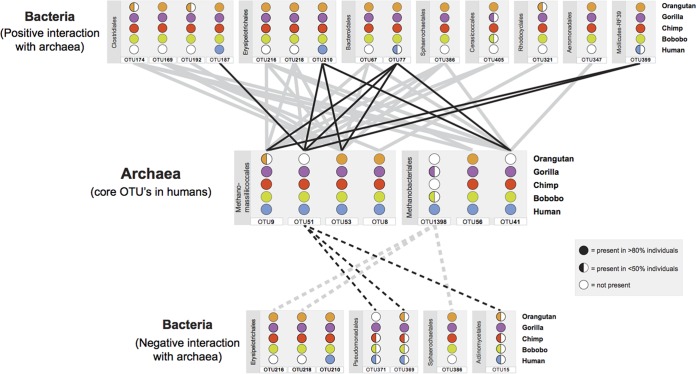
Archaeon-bacterium associations in great ape gut microbiomes. Networks of positive and negative associations between archaeal 94% OTUs and bacterial genera are shown. Associations are based on relative abundances and include only those involving the seven archaeal 94% OTUs comprising the core archaeal microbiome of humans. Distributions of archaeal OTUs and bacterial genera among great ape species are indicated by solid and open circles, respectively. Lines denote statistically significant associations (*P* = 0.001) with a Spearman’s rho value of less than −0.5 or greater than 0.5. Black lines show positive and negative associations between archaeal OTUs and bacterial genera present in humans, whereas gray lines show associations of the same archaeal OTUs with bacterial genera not detected in humans. (See Data Set S5 for additional taxonomic information.)

Because certain archaea in the gut are known to be metabolically dependent on bacteria, we searched for associations between 94% archaeal OTUs and the bacterial genera present in the gut microbiomes of humans and great apes. We considered only those significant negative and positive cooccurrence relationships (*P* < 0.001 after Bonferroni correction) involving the core archaeal 94% OTUs of humans having Spearman’s rho values less than −0.50 or greater than 0.50 with a human- or great ape-associated bacterial genus (see Data Set S5 in the supplemental material). We identified 17 bacterial genera that are significantly positively or negatively correlated with seven of these archaeal 94% OTUs (Fig. 4). Notably, there were no significant associations between the archaeal OTUs assigned as *Methanobrevibacter smithii* (OTU22) or *Methanosphaera stadtmanae* (OTU44), both of which are common constituents in the gut microbiome, and any of the bacterial genera. Overall, we identified more bacterial associations with *Methanomassiliicoccales*-related OTUs than with *Methanobacteriales*-related OTUs: *Prevotella* (OTU77) was found to be positively associated with three *Methanomassiliicoccales*-related OTUs (OTU9, OTU51, and OTU53) and one *Methanobacteriales*-related OTU (OTU41). Most of these significant associations occur between archaeal and bacterial genera that were not present in the human gut microbiome. There were positive associations between *Methanomassiliicoccales*-related OTU53 and *Methanobacteriales*-related OTU41 with *Sphaerochaeta*, *Butyrivibrio*, *Oribacterium*, and unclassified members of the family *Erysipelotrichaceae* and the order *Bacteroidales*, none of which were detected in our human samples (Fig. 4). However, 14 bacterium-archaeon associations that occur in humans are present in multiple great ape species, such as the positive associations among *Methanomassiliicoccales*-related OTUs (OTU9, OTU51, and OTU53) and *Clostridiales* (OTU187), *Erysipelotrichales* (OTU210), *Bacteroidales* (OTU77) and *Mollicutes*-RF39 (OTU399), and between *Methanobacteriales*-related OTU41 and *Bacteroidales* (OTU77) and *Erysipelotrichales* (OTU210) (Fig. 4), and the negative associations between *Methanomassiliicoccales*-related OTU51 and *Pseudomonadales* (OTU369, OTU371) and *Actinomycetales* (OTU15).

10.1128/mSphere.00026-17.5 DATA SET S5  Cooccurrence relationships between archaea and bacteria present in the gut microbiomes of great apes. Only statistically significant associations (*P* = 0.001) with a Spearman’s rho value less than −0.5 or greater than 0.5 are shown. Associations between the nine core 97% OTUs (i.e., those present in all host species) and bacterial genera not detected in human samples are shown in gray. Associations involving these core OTUs and bacterial genera present in humans (and other great apes) are shown in black. Download DATA SET S5, XLSX file, 0.1 MB. Copyright © 2017 Raymann et al. 2017 Raymann et al. This content is distributed under the terms of the Creative Commons Attribution 4.0 International license.

## DISCUSSION

Surveys of the microbial diversity within thousands of individuals (2–6, 16) have led to the view that relatively few archaeal lineages are regular constituents of the human gut microbiome. Until recently, only two methanogenic species, *Methanobrevibacter smithii* and *Methanosphaera stadtmanae*, both in the order *Methanobacteriales*, had been detected in the human gut (1). But the isolation of additional strains representing a new archaeal order, *Methanomassiliicoccales*, demonstrated that other methanogens are occasionally present (17–21). Using primers designed to target archaeal rRNA genes—as opposed to the “universal” prokaryotic primers employed in the majority of studies—we show that previous surveys and strain isolation procedures have vastly underestimated the extent of archaeal diversity in the gut microbiome, even when interrogated at very high sequencing depths.

*Methanobrevibacter smithii* has been reported to occur in 64% to 95% of individuals sampled (22, 23), and several studies suggest that this species is the most abundant methanogen in the human gut and constitutes up to 10% of the anaerobes found in the colon (16, 24, 25). In contrast, *Methanosphaera stadtmanae* and other members of the order* Methanomassiliicoccales* occur in a minority of humans and are present in low abundance (16, 17, 22, 26, 27). Consistent with previous studies, we recovered OTUs representing *M. smithii*, *M. stadtmanae*, and *Methanomassiliicoccales* species in the majority of the humans sampled. Although we sampled a relatively small number of humans from a single geographic location, we observed *M. stadtmanae* and *Methanomassiliicoccales* at much higher frequencies than previously reported, suggesting that these taxa are much more prevalent than currently recognized.

*Archaea* belonging to nonmethanogenic lineages such as halophiles, *Nitrososphaera*, and *Sulfolobus* have also been detected sporadically in the human gut microbiome (2–6, 28–31). Despite the high sequencing depth at which we surveyed *Archaea* (averaging over 40,000 reads per sample), we detected no OTUs belonging to nonmethanogenic lineages: all of the archaeal OTUs we identified in humans fell within the methanogenic orders *Methanobacteriales* and *Methanomassiliicoccales*. Given the limitations of our sampling scheme, it is possible that nonmethanogenic archaea are occasionally present in the human gut microbiome. However, we recovered 120 97% OTUs in humans, over 100 times the number detected in the same samples with universal prokaryotic primers. In addition, 14 of these OTUs were detected in at least 80% of humans, indicating that *Archaea* are prevalent in the human gut.

The numbers of archaeal OTUs in the gut microbiomes of orangutans, gorillas, bonobos, and chimpanzees are high, with each species containing at least twice the 97% OTUs of humans and having representatives of the phylum *Thaumarchaeota*, which were not detected in our human samples. Other studies have identified *Nitrososphaera*, an ammonium oxidizer in the phylum *Thaumarchaeota*, in gut microbiome studies of United States and Malawian populations (1, 5, 29), as well as on human skin (32); therefore, the lack of detection of *Thaumarchaeota* in our human samples could be due to sampling bias. Due to their sporadic occurrence and relatively low frequencies in gut microbiomes, *Thaumarchaeota* could be transitory and not habitual gut constituents. The fact that 74 of the 97% OTUs from the orders *Methanobacteriales* and *Methanomassiliicoccales* were detected in all great ape species, and nine of these shared OTUs are present in the majority of individuals in each species, suggests that these lineages occurred in the ancestor of all great apes and have possibly coevolved with their hosts for millions of years, as reported for some bacterial lineages (33).

By studying the gut microbiome great apes in a phylogenetic context, we can begin to gain insight into how the composition of the gut microbiome has changed over time. Analysis of humans, chimpanzees, bonobos, gorillas, and orangutans reveals that there has been a loss of archaeal diversity during the diversification of great apes, particularly in the human lineage. Because methanogens help degrade plant polysaccharides by using the end products of bacterial fermentation (17–21, 34), and some methanogens are capable of reducing methanol, which is generated through bacterial degradation of pectin, mainly from fruits (22, 23, 35), the higher archaeal diversity in nonhuman apes could reflect their plant-based diets: gorillas and orangutans, which are principally herbivorous, have a higher level of archaeal diversity than do omnivorous chimpanzees and humans. Although recent studies have shown that captivity can cause reductions in bacterial diversity of gut microbiomes (36, 37), the orangutans, which were all sampled from a zoo colony, averaged the highest level of archaeal diversity of all great apes—even higher than that of gorillas, chimpanzees, and bonobos, which were sampled from wild populations. Moreover, our surveys of bacterial diversity using universal prokaryotic primers revealed that orangutans averaged the highest level of bacterial diversity as well. Since microbiome reductions are often ascribed to dietary restrictions of plant material, the maintenance of high levels of diversity in captive orangutans likely reflects their diverse plant-based diet, which includes more than a dozen families of fruits, grains, roots, and leafy vegetables. Nutrients from fruits, stems, and leaves are fermented in the colon, which is much larger in nonhuman apes—the human colon occupies 17 to 23% of the digestive tract, whereas in chimpanzees, orangutans, and gorillas it is 52 to 54% (38). This reduction in fermentation-assisting archaea, like colon size, could be a response to higher-quality foods, which can be digested in the small intestine (38).

In many environments, including the human gut, archaea live in syntrophy with bacteria (39), and there are numerous archaeal OTUs whose presence or absence coincided with particular bacterial genera in the microbiomes of great apes. We identified positive associations of the bacterial genus *Prevotella* (OTU77 in Fig. 4) with *Methanobrevibacter* (OTU41) and* Methanomassiliicoccales* (OTU9, OTU51, and OTU53). OTUs in *Methanobrevibacter* and *Prevotella* were previously assigned as members of distinct gut enterotypes (40), so their cooccurrence in all great ape species was not anticipated, although later studies have reported positive associations between these taxa (29, 41). Most of the bacterium-archaeon associations that we identify have never previously been observed, but that is because most involve bacterial taxa not present in human samples. This suggests that along with the decrease in archaeal and bacterial diversity in the human gut microbiome, many associations between bacteria and archaea have been lost. However, several bacterium-archaeon associations are maintained in multiple great ape species, suggesting evolutionary conservation and providing a starting point for future investigations on the role of archaea in the gut microbiome.

Here, we show that much of the archaeal diversity in the gut microbiome has been overlooked due to the reliance on prescribed sets of universal prokaryotic primers to characterize microbiomes. High numbers of OTUs were detected despite the limited numbers of sites and samples that were examined for each species, suggesting that all great apes possess extensive species-level diversity of *Archaea* in their gut microbiomes. Nearly all *Archaea* we identified in great apes belong to the orders *Methanobacteriales* and *Methanomassiliicoccales*; therefore, they are all likely hydrogen-utilizing methanogens. The few hydrogen-utilizing methanogens (*M. smithii*, *M. stadtmanae*, *Methanomassiliicoccus luminyensis*, “*Candidatus* Methanomassiliicoccus intestinalis,” and “*Candidatus* Methanomethylophilus alvus”) already known to occur in the human gut are capable of using different or multiple substrates for methanogenesis, including carbon dioxide, methanol, or the methyl compounds monomethylamine, dimethylamine, and trimethylamine (1). Because methanogens compete for hydrogen in the gut, it is possible that some taxa are capable of performing metabolic capabilities that allow them to interact with different hydrogen-producing organisms and maintain distinct niches. Although we observed a decrease in archaeal diversity in the human lineage, we were able to identify several archaeal OTUs that are conserved across all great ape species, and their persistence implies a continual role in the breakdown of dietary compounds throughout hominoid evolution.

## MATERIALS AND METHODS

### Sample sources and DNA extraction procedures.

Samples representing five great ape species were obtained from the following sources. (i) For humans, DNAs were purified from fecal samples of 10 individuals following the method of Shannon et al. (42). (Sample sources and designations are presented in Data Set S3.) (ii) For chimpanzees, DNAs, purified from field-collected fecal samples from 15 individuals (*Pan troglodytes*), were selected from those reported in reference 6. DNA extraction procedures and bacterial contents of samples, as surveyed with 16S rRNA gene universal prokaryotic primer pair 515F/806R, are also presented in reference 6. (Sample sources and designations are presented in Data Set S3.) (iii) For gorillas, DNA samples, purified from field-collected fecal samples from 20 individuals (*Gorilla gorilla*), were selected from those reported in reference 6. DNA extraction procedures and bacterial contents of samples, as surveyed with 16S rRNA universal prokaryotic primer pair 515F/806R, are also presented in reference 6. (Sample sources and designations are presented in Data Set S3.) (iv) For bonobos, DNA samples, purified from the field-collected fecal samples from 18 individuals (*Pan paniscus*) were obtained from Moeller et al. (6). (Sample sources and designations are presented in Data Set S3.) (v) For orangutans, fecal samples were obtained from 10 individuals (*Pongo pygmaeus*) from a colony at the Atlanta Zoo. (Sample sources and designations are presented in Data Set S3.) Upon collection, each sample was mixed with an equal volume of RNAlater (Ambion) and stored frozen at −80°C. Total DNA from each fecal sample was extracted from 400-mg aliquots following the bead-beating procedure described in reference 43.

### Sample preparation and DNA sequencing.

DNAs were subjected to PCR amplification targeting the V4-V5 variable region of archaeal 16S rRNA genes using the primer set Arch516F/Arch915R (8, 9). Three replicate PCRs were performed on each sample, and the amplicons generated from each set of three reactions were pooled and quantified on a Qubit 2.0 fluorometer (Invitrogen). Replicate samples were pooled and individually bar coded according to procedures outlined in reference 10. Amplicons were purified with AMPure XP beads (Beckman Coulter, Inc.). Bar-coded samples were pooled and sequenced on the Illumina MiSeq at the Genome Sequencing and Analysis Facility at the University of Texas at Austin.

Sequencing data corresponding the V4 region, as amplified with universal prokaryotic primers 515F/806R, were obtained from published sources: data for gorillas, bonobos, and chimpanzees were from reference 6. Because samples from humans and orangutans had not previously been characterized for microbial diversity with the universal prokaryotic primer set, PCR amplifications of the corresponding V4 region of 16S rRNA were performed on these samples with the 515F/806R primer pair using the protocol described above.

### Sequence filtering and analysis.

Sequence reads from the universal prokaryotic primer data sets and the archaeon-specific primer data set were processed in QIIME (44). For the *Archaea*-specific primer data set, FASTQ files were quality filtered with split_libraries_fastq.py, allowing a minimum Phred score of Q20. Forward and reverse Illumina reads were joined with join_paired_ends.py. Chimeric sequences, identified using USEARCH6.1 implemented in the identify_chimeric_seqs.py script in QIIME, were removed (3% of the total reads). OTUs were clustered at both 94% sequence identity and 97% sequence identity by UCLUST, as implemented in pick_open_reference_otus.py. Additionally, OTUs were clustered using VSEARCH (43). On average, the VSEARCH clustering method detected slightly higher numbers of OTUs than UCLUST (Tables S1A and B), so to be conservative, all subsequent analyses were performed on OTUs clustered by UCLUST. Sequence reads were initially searched against the July 2015 release of the SILVA 94% and 97% reference database (http://www.arb-silva.de/download/arb-files) (11). Sequences that did not find matches in the SILVA database at 94% or 97% were subsequently clustered into *de novo* OTUs with UCLUST. All OTUs represented by <10 reads were removed, as were any reads that matched mitochondrial or chloroplast sequences and those that could not taxonomically assigned to *Bacteria* or *Archaea* (“unassigned reads”). A reference sequence from each archaeal OTU (clustered at 97% and at 94% sequence identity) was selected and aligned using PyNAST (10). This alignment was used to construct a phylogenetic tree using FastTree (45) within QIIME. To compare the extent of archaeal diversity captured with universal prokaryotic primers to that detected with archaeon-specific primers, the data set was analyzed both with and without bacterial reads. Downstream analyses, including the taxonomic assignment of OTUs, and estimates of alpha and beta diversity were conducted using the QIIME workflow core_diversity_analysis.py, applying a sampling depth of 10,000 reads per sample and default parameters. Rarefaction depths were chosen manually to exclude samples with low total sequences (see Data Set S2 for sample details). The final sample sizes for each species after rarefaction were as follows: *Homo sapiens*, *n* = 10; *Pan troglodytes*, *n* = 14; *Pan paniscus*, *n* = 18; *Gorilla gorilla*, *n* = 20; and *Pongo pygmaeus*, *n* = 8.

For the data set generated with universal prokaryotic primers, the FASTQ files for the 10 humans, 14 chimpanzees, 18 bonobos, 20 gorillas, and 8 orangutans were filtered for quality with split_libraries_fastq.py, allowing a minimum Phred score of Q20. Forward and reverse Illumina reads were joined with join_paired_ends.py, and the joined paired reads were trimmed to equal lengths. Chimeric sequences, identified using USEARCH6.1 implemented in the identify_chimeric_seqs.py script in QIIME, were removed (amounting to 1.5% of total reads). OTUs clustered at 97% sequence identity were chosen by UCLUST, as implemented in pick_open_reference_otus.py. Sequence reads were initially searched against the July 2015 release of the SILVA 97% reference database (http://www.arb-silva.de/download/arb-files) (11). Sequences that did not find matches in the SILVA data set were clustered into *de novo* OTUs with UCLUST. All OTUs represented by less than 10 reads were removed, as were any reads that matched mitochondrial or chloroplast sequences and those that could not be taxonomically assigned to *Bacteria* or *Archaea*. Downstream analyses, including taxonomic classification of OTUs and estimates of alpha and beta diversity, were conducted in the QIIME workflow core_diversity_analysis.py, applying a sampling depth of 10,000 reads per sample and default parameters. Statistical tests and boxplots were completed in R, and Venn diagrams using the tool from http://bioinformatics.psb.ugent.be/webtools/Venn/. Great ape divergence times follow those reported in reference 46. The coverage and specificity of the primers used in this study were evaluated with TestPrime 1.0 implemented on the SILVA database (Data Set S1) (47).

### Phylogenetic analysis.

The 16S rRNA sequences from 58 complete archaeal genomes were obtained from the RDP (13), combined with reference sequences from each archaeal OTU, and aligned with MUSCLE v3.8.31 (48). Sequences were trimmed manually in SeaView (49), and a maximum likelihood tree was inferred with PhyML v3.1 using the GTR+Γ4 model (50). The reliability for the internal branches of the maximum likelihood trees was assessed by bootstrapping and the approximate likelihood ratio test (aLRT–SH-Like) (51).

### Association networks and correlation analysis.

We tested the associations between 102 archaeal OTUs (clustered at 94%) and the 415 bacterial genera (clustered at 97% and classified to genera using the RDP classifier) using Spearman’s rank correlation (rho) implemented in R. We extracted statistically significant (*P* < 0.001, after Bonferroni correction) negative and positive cooccurrence relationships with Spearman’s rho values less than −0.50 or greater than 0.50 that involved archaeal OTUs present in >80% of all human samples. Association networks were visualized with Cytoscape (42).

### Ethics statement.

The study protocol was approved by the Yale University Human Investigation Committee. Informed consent was obtained from all human subjects as described in our approved protocol no. 1106008725.

### Accession number(s).

All sequence data have been deposited into the NCBI Sequence Read Archive under accession no. PRJNA371799 and PRJNA338781.
