# Supplementary material for: Unexplored Archaeal Diversity in the Great Ape Gut Microbiome
Source: mSphere. 2017 Feb 22;2(1):e00026-17. doi: 10.1128/mSphere.00026-17 (PMC5322346; doi:10.1128/mSphere.00026-17)
Supplement: TABLE S1 [file sph002172239st10.pdf]

Table S1 A. Number of OTUs detected with VSEARCH

Species	Total OTUs @ 10,000 reads	Mean OTUs @ 10,000 reads	SD
Orangutan	297	182	22.59
Gorilla	393	163	42.17
Chimpanzee	253	92	28.83
Bonobo	266	96	32.81
Human	140	49	14.36

Table S1 B. Number of OTUs detected with UCLUST

Species	Total OUT @ 10,000 reads	Mean OTUs @ 10,000 reads	SD
Orangutan	302	161	18.55
Gorilla	470	135	36.15
Chimpanzee	235	69	18.67
Bonobo	247	71	24.29
Human	120	37	10.16
