# Supplementary figures and images for: Unexplored Archaeal Diversity in the Great Ape Gut Microbiome
Source: mSphere. 2017 Feb 22;2(1):e00026-17. doi: 10.1128/mSphere.00026-17 (PMC5322346; doi:10.1128/mSphere.00026-17)

**A**

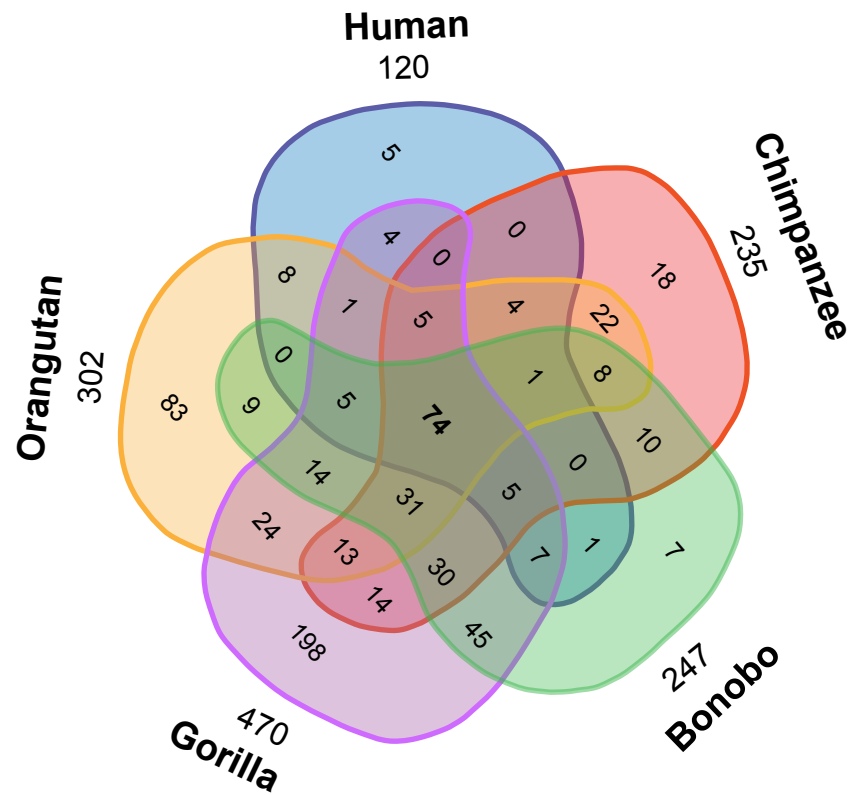

**B**

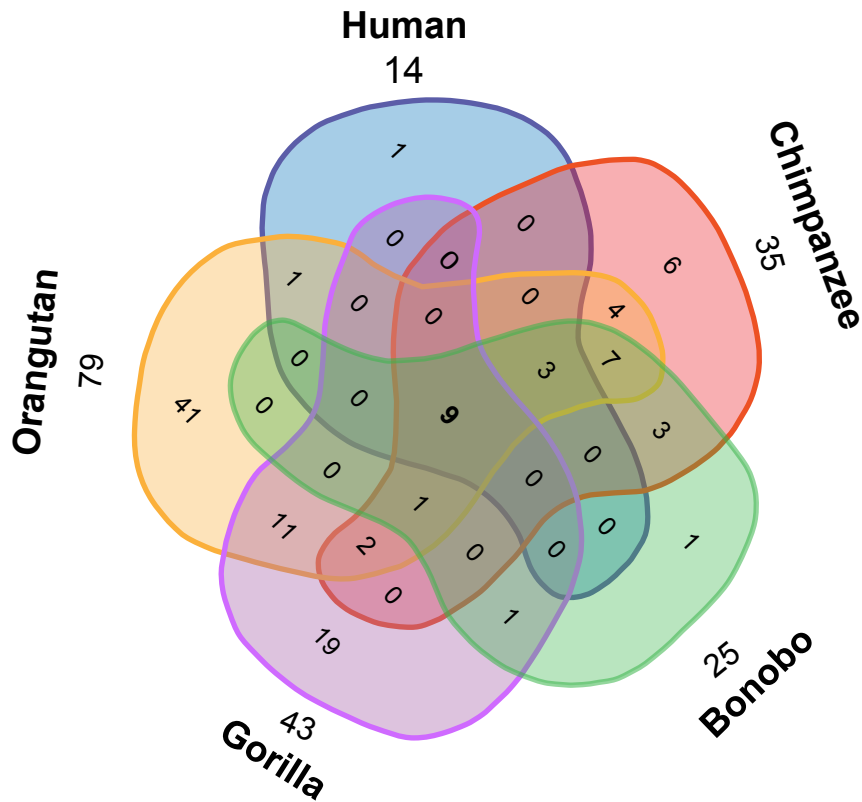

Supplement: FIG S1 [file sph002172239sf6.pdf]

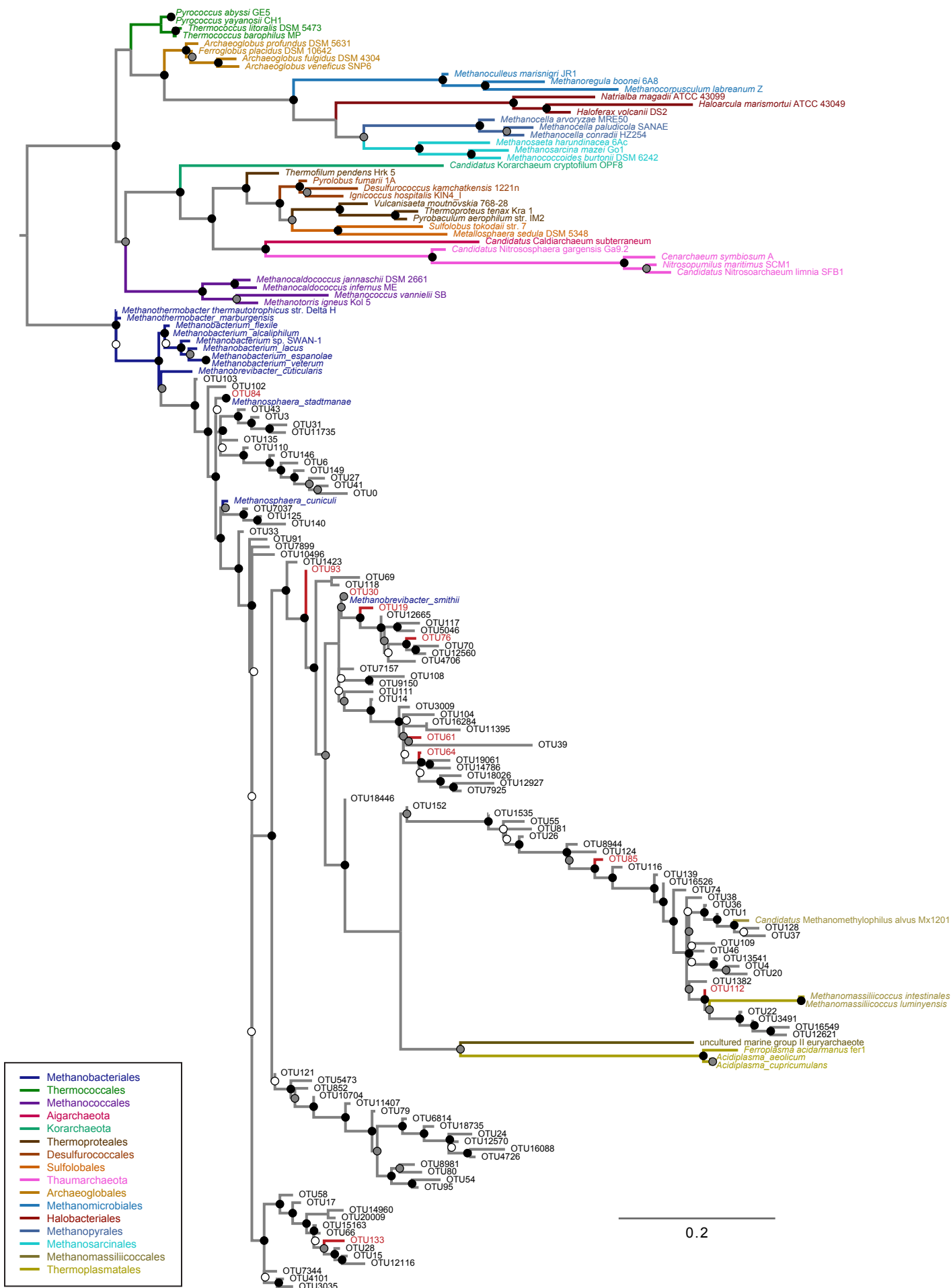

Supplement: FIG S2 [file sph002172239sf7.pdf]

**A**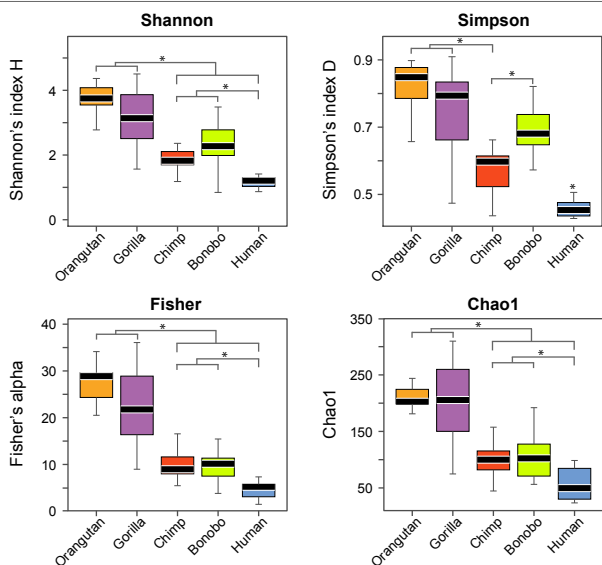**B**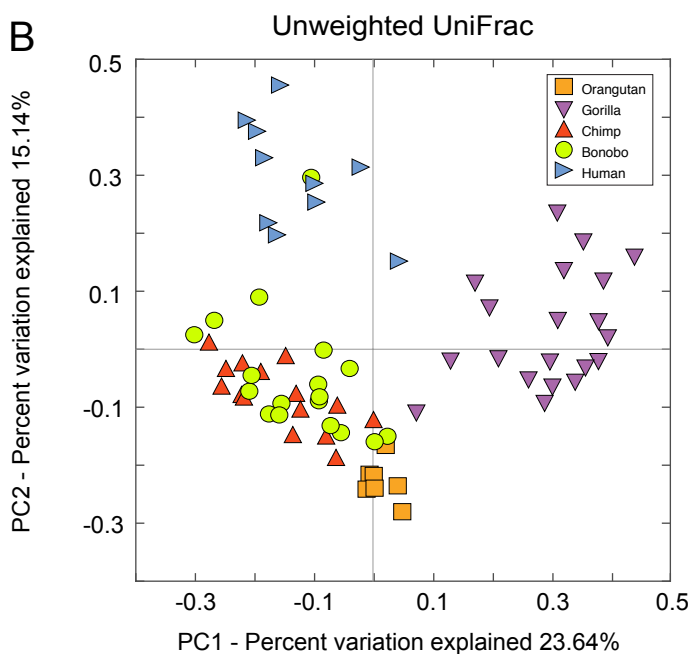**C**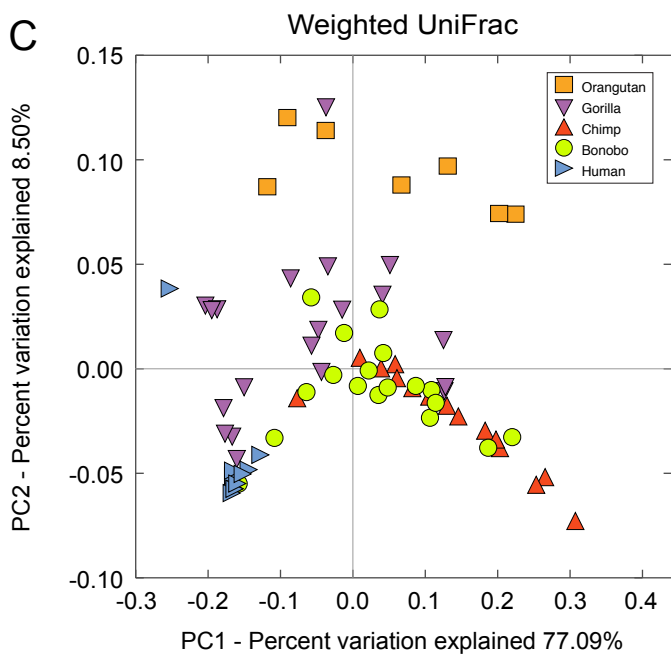

Supplement: FIG S3 [file sph002172239sf8.pdf]

# ARCHAEAL 94% OTUs

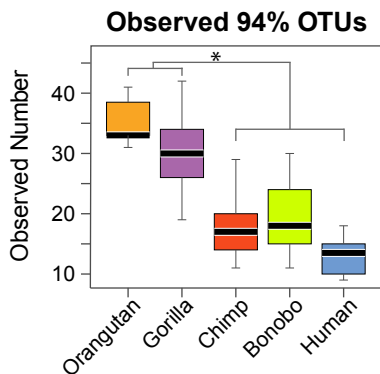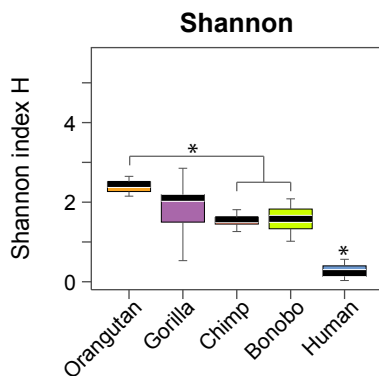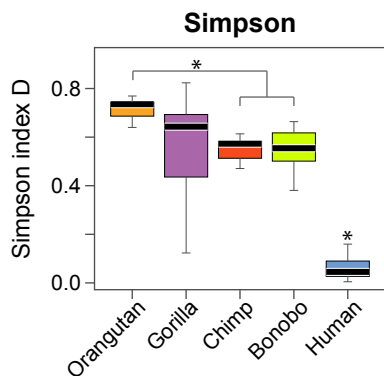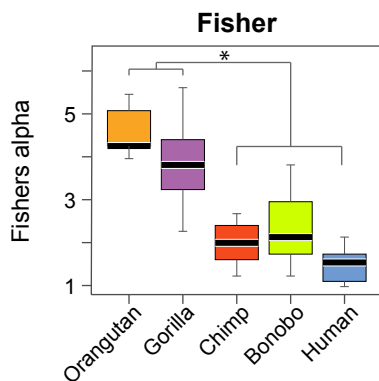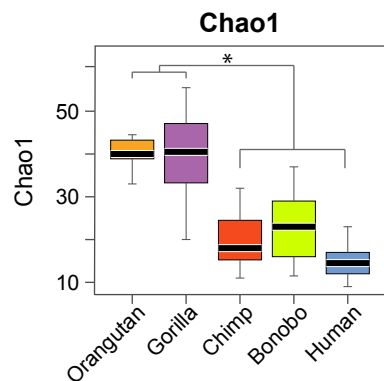

Supplement: FIG S4 [file sph002172239sf9.pdf]
